# Sub-zero temperature mechanically stable low molecular weight hydrogels†
†Electronic supplementary information (ESI) available: Full experimental procedures, rheology and supplementary images. See DOI: 10.1039/c8tb01668b


**DOI:** 10.1039/c8tb01668b

**Published:** 2018-08-02

**Authors:** Alice E. R. Fayter, Matthew I. Gibson, Emily R. Draper

**Affiliations:** a Department of Chemistry , University of Warwick , CV4 7AL , UK; b Warwick Medical School , University of Warwick , CV4 7AL , UK; c School of Chemistry , University of Glasgow , Glasgow , G12 8QQ , UK . Email: Emily.Draper@glasgow.ac.uk

## Abstract

We show here a low molecular weight hydrogelator based on a functionalised-dipeptide which is stable down to temperatures of –12 °C despite being made from >99% water. This stabilty at low temperature can be extended to ∼–40 °C by gelling water : glycerol mixtures. The temperature range is wider than that of the glycerol : water mixtures alone. The rheological properties of the gels do not change at this low temperature compared to that of gels at 25 °C. This freezing point depression offers a potentially new method of transporting gels and offers the prospect of hydrogels being used at much lower working temperatures whilst retaining the desired rheological properties, this is useful for cryopreservation.

## Abstract 2

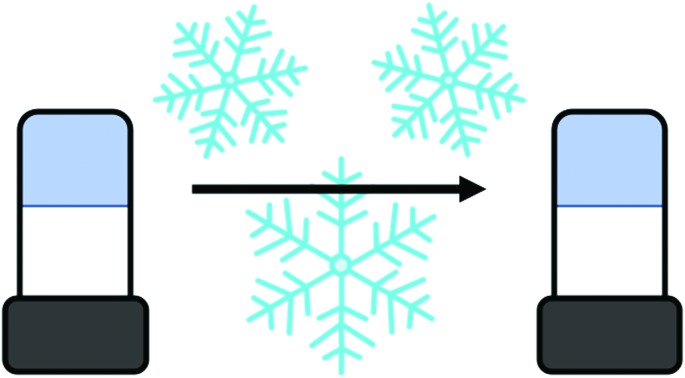
We show here a low molecular weight hydrogelator based on a functionalised dipeptide which is stable down to temperatures of –12 °C despite being made from >99% water. With the addition of glycerol this can be lowered further to –40 °C. At these colder temperatures there is no effect on the mechanical properties of the gels.

## Introduction

Freezing-point depression of water is useful when low working temperatures are needed, for example in cryopreservation of bacteria, mammalian cells and enzymes.^1^–^3^ All of these can be damaged by high temperatures, so storage at low temperatures is desirable. However, they can also be damaged by the formation of ice crystals, so the inhibition of these ice crystals is needed.^2^,^4^ Other situations where low temperature stability is also essential include when using smart materials in an uncontrolled temperature setting, such as windows on the outside of buildings, which go through various temperature changes throughout the day and year.^5^ Some analytical methods, such as Dynamic Nuclear Polarisation (DNP) NMR work at very low temperatures, so if one wanted to analyse something in solution such as a hydrogel, this would be almost impossible without a freezing point suppressant. Decreasing the freezing point can be achieved by using freezing depressant additives such as salts, glycerol, sorbitol, glycoproteins, or organic solvents with low freezing points.^6^ However, the addition of some additives is detrimental to biological samples or can completely change the properties of the water they are in, and so completely change the sample.^7^–^9^

Low molecular weight gels (LMWGs) are a class of material with interesting and diverse range of properties and have been used with biological samples such as in cell culture and drug delivery.^10^–^13^ In the case of hydrogels, they can be made from >99% water, with less than 1% of material self-assembling into long fibrous structures that entangle and trap the water. It is these long gel fibres which can be used as artificial extracellular matrices but also as conductive fibres, depending on what they are made from.^14^,^15^ They are now finding uses in water purification,^16^ solar fuel cells, electronic devices, actuators *etc.* all of which will be subject to a range of working temperatures.

There are many examples of the effect of heat on these gels to either form the gels *via* a heat–cool trigger, to control the supramolecular structures formed from the LMWGs and so control morphology of the gel fibres or behaviour of the gel properties.^17^–^20^ Melting of the gels can also be used to determine gel fibre composition in multicomponent gel systems.^21^,^22^ There are however very few examples of these gels at low temperatures, apart from to the control the kinetics of gelation.^23^ Berillo *et al.* looked at gelling a Fmoc-Phe-Phe gelator in water at –12 °C with and without salt present.^24^ They found gels formed in the cold temperatures were less mechanically strong than ones formed at room temperature. In polymer systems, the upper/lower critical solution temperatures (U/LCST) are often considered as their phase behaviour (solubility) is modulated by the external temperature but this is rarely discussed for LMWGs.^25^ Polymer gels can be used as actuators and can swell, move and even change shape in response to an increase in temperature.^26^ There are many examples of temperature stable polymer gels using PVA with or without glycerol present in the systems.^27^–^30^ In anti-freezing polymer gel systems, the water is often replaced entirely with a solvent with a lower freezing point or an additive added into the water. For example, recently Zhou and co-workers showed an organohydrogel based on a Ca-alginate/polyacrylamide blend where they replaced water with either glycol, sorbitol or glycerol and showed stability of the gel down to –70 °C.^31^ However, the shape and the mechanical properties were dramatically altered by this process and so the original properties of the gel were not retained. Since a significant amount of the water was replaced with the additive, the biocompatibility would also be different to the original gel. LMWGs would be expected to be less tolerant to these cold temperatures as they are held together by weak non-covalent bonding compared to polymer gel systems, and they generally contain less structuring materials. The formation of ice would logically be expected to destroy the LMWG network, as opposed to making the polymers gels more mechanically ridged or sometimes more fragile.^32^

**Fig. 1:**
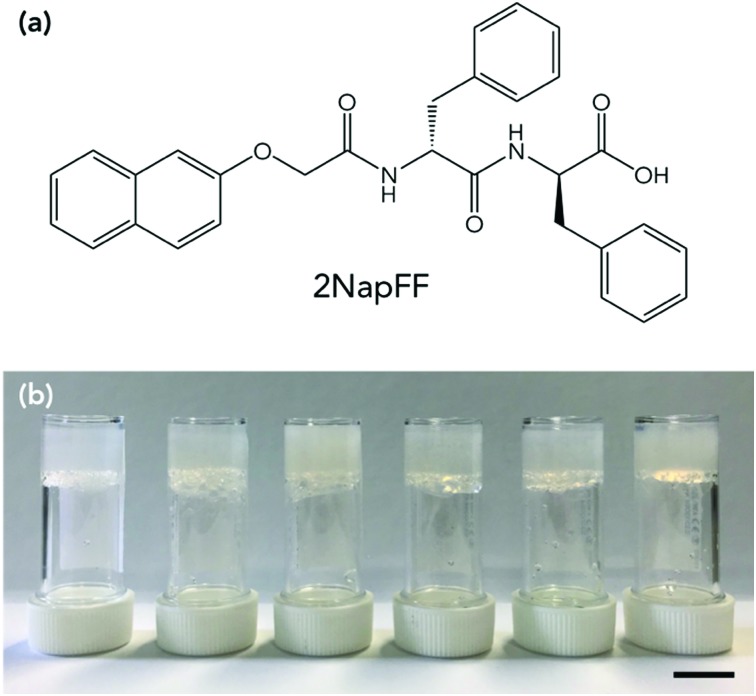
(a) Chemical structure of gelator 2NapFF. (b) Photographs of 2NapFF at (left to right) 10 mg mL^–1^, 5 mg mL^–1^, 2.5 mg mL^–1^, 20 : 80 glycerol : water (5 mg mL^–1^), 40 : 60 glycerol : water (5 mg mL^–1^) and 60 : 40 glycerol : water (5 mg mL^–1^). Scale bar is 1 cm.

Here, we use a LMWG based on a dipeptide, which we refer to as 2NapFF throughout (Fig. 1a). We gel 2NapFF in water at different concentrations and assess the stability at low temperatures by monitoring the rheological properties and by microlitre nucleation measurements to separate heterogeneous nucleation events. We then use glycerol as an additive to improve the properties of the gels at low temperatures.

## Results and discussion

2NapFF solutions were prepared at 2.5, 5 and 10 mg mL^–1^. The gelator was dissolved in water by the addition of one molar equivalent of NaOH and made up to the correct volume with distilled water. The samples were stirred overnight until all the gelator had dissolved. This resulted in a viscous transparent solution at pH 9.^33^,^34^ In the case of the of the glycerol:water solutions, these were all prepared at a concentration of 5 mg mL^–1^ of 2NapFF. The solutions were prepared as described above but the water replaced with 20 : 80, 40 : 60 and 60 : 40 glycerol : water by volume (higher volumes of glycerol did not result in a gel).

A slow acidification method was used to gel the solution. This was achieved by adding 8 mg mL^–1^ of glucono-δ-lactone (GdL) per 5 mg of gelator in solution.^35^ The GdL was gently mixed in the solutions by hand to ensure dissolution, and then the samples left untouched overnight to result in self-supporting gels with a pH of around 3.3 (Fig. 1b). Gels were prepared in aluminium cups for rheological measurements to ensure efficient heat transfer from the rheometer to the gels. The effect of gelator concentration on freezing point was investigated. Each of the gels was first characterised by rheological strain and frequency sweeps at 25 °C (Fig. S1, ESI†). The gels were reproducible and each yielded at low strains (between 5–10%), flowed at higher strain (>100%) and varied in storage modulus (*G*′) and loss modulus (*G*′′) depending on gelator concentration, with 10 mg mL^–1^ being the stiffest and 2.5 mg mL^–1^ the softest. The concentration affected the strain behaviour as well as *G*′ and *G*′′. The gel at 2.5 mg mL^–1^ was found to have essentially a single fracture break (Fig. S1a, ESI†), whereas 5 and 10 mg mL^–1^ showed multiple yield points before flowing (Fig. S1c and e, ESI†). 2NapFF structures in the gel phase have been shown to be dependent on concentration previously, explaining the different strain behaviours.^33^,^34^ All the gels showed behaviour that was independent of frequency. Adams and co-workers have previously studied the effect of concentration on 2NapFF GdL gels. They found that at all concentrations the morphology and gel fibres were very similar, and the differences in *G*′ and *G*′′ were a result in density of the fibres present, rather than a different gel network or fibre morphology.^34^

**Fig. 2:**
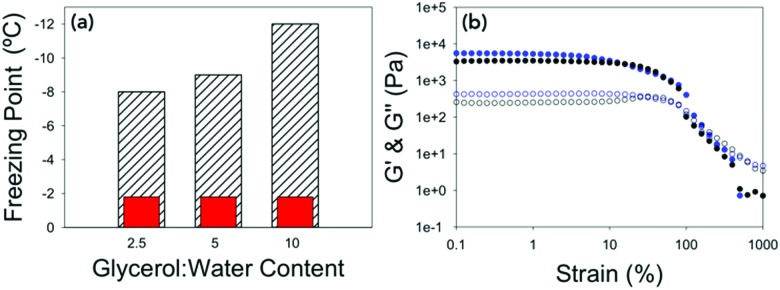
(a) Bar chart showing the expected freezing point of the water *vs.* the freezing point of the gels at different concentrations of 2NapFF. Hatched bars are the measured freezing point and red bars are the freezing point of distilled water on the rheometer (Fig. S2d, ESI†). (b) Strain sweeps performed at 10 rad s^–1^ for 2.5 mg mL^–1^ 2NapFF at 25 °C (black data) and at –7 °C (blue data). In both graphs, *G*′ is the closed shapes and *G*′′ is the open shapes. No error bars included for clarity.

The temperature stability of the gels was then determined. This was done by lowering the temperature of the gel at a rate of 0.5 °C min^–1^ at 10 rad s^–1^ and 0.5% strain (within the linear viscoelastic region (LVR) of the gel as determined from the previous measurements). The freezing point was determined by the point at which *G*′ and *G*′′ dramatically increased in value due to ice crystals being formed and the sample becoming a solid (Fig. 2a and Fig. S2, ESI†). All of the gels measured showed very little change in mechanical properties until the gel froze. This is seen by there being no change in tan *δ* when the temperature is lowered until the gel freezes and tan *δ* changes dramatically. The freezing point of the gels were depressed in line with the concentration of gelator in the gel, with 10 mg mL^–1^ having a freezing point of –12 °C, 5 mg mL^–1^ a freezing point of –9 °C and 2.5 mg mL^–1^ a freezing point of –8 °C. What is most remarkable is that the rheological properties of the gels just before the freezing point were the same as if they were at room temperature (Fig. 2b and Fig. S3, ESI†) showing despite changing the temperature dramatically, the mechanical properties of the gels remain the same. The difference in the freezing points could be due to there being more organic material in the gel, which suppresses the freezing point by colligative effects. Alternatively, the increased amount of organic material may result in a denser network leading to segregation of any ice crystals which do nucleate, preventing them spreading. The gel fibres could also be acting as a freezing point suppressant in a similar way to glycerol by having an extended hydrogen bonded network which changes the hydrogen bonding between the water molecules.^36^

**Fig. 3:**
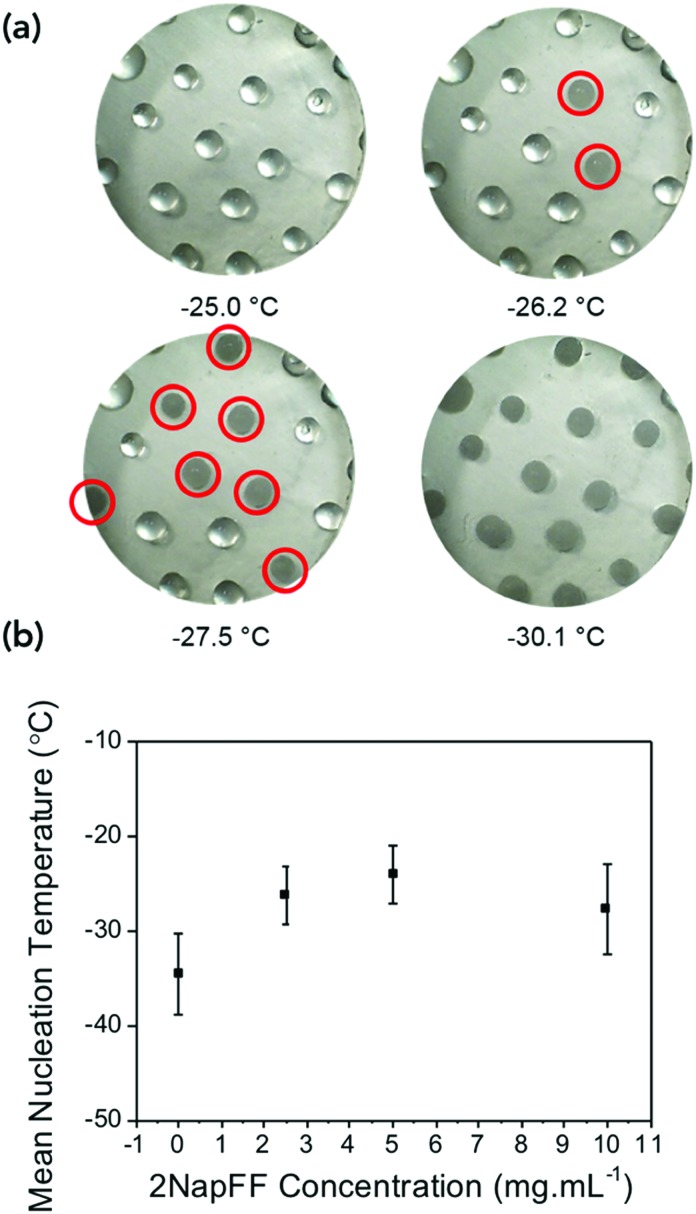
Ice nucleation assay. (a) Example multi-point freezing assay used to assess the nucleation temperature as the temperature is reduced. Nucleating droplets are circled in red. (b) Mean nucleation temperature as a function of gelator concentration.

The differences in the frozen gels and unfrozen gels could be clearly seen by eye. The frozen gels would stick to the geometry of the rheometer and were opaque whereas the unfrozen gels (still at a cold temperature) remained transparent and soft (Fig. S4, ESI†). To investigate if the gels were inhibiting the heterogeneous nucleation of ice (the most common form of ice nucleation due to impurities) a microlitre ice nucleation assay was employed. As nucleation is a stochastic process, small droplets are essential to reduce the number of unwanted nucleators, and a large number of repeats are necessary as the individual nucleator temperatures will always vary.^37^Fig. 3 shows example freezing of microlitre drops of set gels as a function of temperature in a cryo-microscope, with freezing identified by the droplets becoming cloudy. In this system pure water showed a homogeneous freezing point of ∼–35 °C, as expected taking into account some thermal gradients in the system. 2NapFF gels showed heterogeneous nucleation temperature of –20 to –28 °C as the concentration increased (Fig. 3b). These values are lower than the bulk, as the mechanical action of the rheometer will promote ice nucleation in super-cooled water.

Another method of suppressing the freezing point is to add a cosolvent into the water. The additive would need to be miscible with water and not affect the gelation ability of 2NapFF. A gelator concentration of 5 mg mL^–1^ was used to examine the addition of glycerol into the water. Glycerol is known to lower the freezing point of water^38^ and is widely used a cryoprotective agent in microbiology.^39^ Different ratios of glycerol to water can be used to tune the freezing point of the water, and therefore the gel. Ratios of 20 : 80, 40 : 60, 60 : 40 and 80 : 20 glycerol : water were compared to the data for 0 : 100 described above. The 80 : 20 mixture did not result in gelation, but gelation occurred in the other mixtures. Gelation of the glycerol : water mixtures using the 2NapFF were reproducible, with gels at 20 : 80 and 40 : 60 having comparable rheological properties to that of water-only gels at the same concentration (Fig. S5a–d, ESI†).

This suggests that the glycerol is not having a significant effect on the gelation process. The gels at 60 : 40 had a slightly lower *G*′ and *G*′′ value than the other gels but have a similar strain behaviour (Fig. S5e and f, ESI†).

**Fig. 4:**
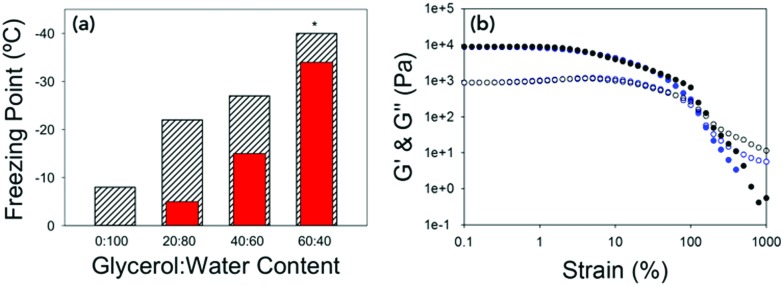
(a) Bar chart showing the expected colligative freezing point of the water *vs.* the freezing point of the gels at different ratios of glycerol : water. Hatched bars are the measured freezing point and red bars are the expected freezing points.^38^ * For 60 : 40 the freezing point was not reached, but is beyond –40 °C. (b) Strain sweeps performed at 10 rad s^–1^ for 40 : 60 at 25 °C (black data) and at –25 °C (blue data). In both graphs *G*′ is the closed shapes and *G*′′ is the open shapes. No error bars included for clarity.

Again, the temperature dependence and freezing points of the glycerol : water gels were determined by keeping a constant strain and frequency and lowering the temperature until the gels froze (Fig. 4a and Fig. S6, ESI†). For the gel prepared at 20 : 80, the freezing point was –22 °C, the gel at 40 : 60 –27 °C and the gel at 60 : 40 did not freeze at –40 °C, which is the lowest temperature to which the rheometer is able to achieve. Interestingly the freezing points are lower than the expected colligative freezing point values of water and glycerol mixtures.^40^ For glycerol : water mixtures, a mixture of 20 : 80 should freeze at –5 °C, 40 : 60 at –15 °C and 60 : 40 at –34 °C.^38^ This suggests that the 2NapFF and the glycerol are acting synergistically to reduce the freezing point of the gels. Compared to the freezing point of the 100% water gel of –9 °C, this a dramatic increase in the freezing point with little to no change to the rheological properties. The strain sweeps were then conducted a few degrees above the freezing point (Fig. 4b and Fig. S7, ESI†). The gels prepared at 20 : 80 and 40 : 60 have almost identical rheological properties to the gels prepared at 25 °C, showing that the cold temperature has no effect on the mechanical properties of the gels.

**Fig. 5:**
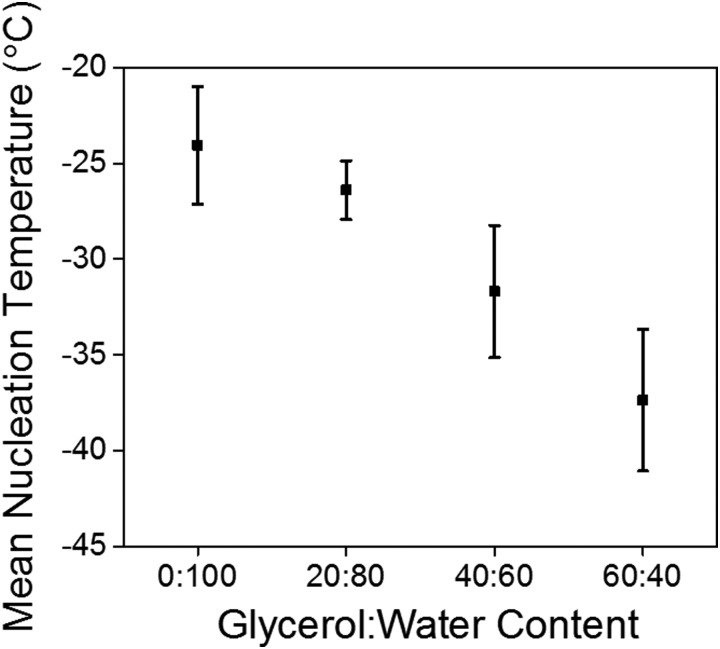
Mean nucleation temperature of LMWG formed in presence of glycerol.

The gels could be chilled at a temperature above the freezing point and held at that temperature before being returned to room temperature and again the mechanical properties are unaffected (Fig. S8, ESI†). However, if the gel was allowed to freeze and then warmed back up the gel had been damaged and was now significantly changed mechanical properties due to the network being damaged due to ice-crystal formation (Fig. S9, ESI†). Microlitre nucleation assays were again used to probe the nucleation temperature of glycerol-containing gels, (Fig. 5 and Fig. S9, ESI†). Increasing glycerol concentration as to 60 : 40 reduced the nucleation temperature to –38 °C, which agreed with the rheology data confirming the depression of the freezing point is due to colligative effects.

Next, we wanted to look whether other dipeptide gelators exhibited the same behaviour. We looked at gelators that formed different structures at high pH, had very different chemical structures, and also examples that had similar aggregation at high pH to 2NapFF (Fig. S10, ESI†). These included 2NapVG, which doesn’t form aggregates at high pH,^41^ PBI-H which forms worm-like micelles at high pH but has a very different chemical structure,^42^ ThFF which has a similar chemical structure and forms aggregated structures at high pH^43^ and ArFF which has a very similar chemical structure and also exhibits the same behaviour to 2NapFF after heating and cooling.^17^

All these samples were prepared at 5 mg mL^–1^ of gelator with 20 : 80 glycerol : water, and 8 mg mL^–1^ of GdL was used to trigger gelation. For ThFF, PBI-H and 2NapVG, these all had freezing points of around –12 °C, and so lower than that of 2NapFF with 20 : 80 glycerol : water, but still lower than expected from the glycerol content (Fig. S11 and S12, ESI†). However, ArFF had a freezing point of –20 °C, similar to that of 2NapFF. This suggests that the freezing point depression is not to do with the molecular structure of the LMW gelators, but rather due to the increased viscosity. As viscosity increases the diffusion constant for the water molecules reduces, leading to a lengthening of diffusional mixing time, thus there is a larger barrier to molecular rearrangements within the sample hindering the formation of a critical nucleus causing the nucleation temperature to decrease.

## Conclusions

We have been able to show that the freezing point of hydrogels can be significantly depressed by either changing the concentration of gelator, or by gelling a glycerol : water mixture. The freezing point can also be tailored by changing the amount of glycerol present in the gel. The amount that the freezing point is depressed is more than that of glycerol : water mixtures alone can achieve and so shows that the gelator network is acting synergistically with the glycerol to prevent the formation of ice crystals. This offers the exciting possibility of using these gels not only at ambient conditions, but also at more extreme conditions. This is normally done using organogels with organic non-biocompatible solvents with low freezing points or using polymer systems which require a lot of processing to achieve this temperature stability. These results also potentially open up the opportunity of enabling easier transporting or handling as gels are less likely to dry out at the colder temperatures and/or with the addition of glycerol. In addition, these cold gels could be used to store and transport enzymes and other biological tissues as a means of cryopreservation. There is also the potential for these gels to be used to study kinetics within gels where processes are slowed down making them easier to follow, for them to be used in smart technologies where they are used outside where there is often a more demanding temperature requirement than in the laboratory, and also possibly in techniques such as DNP NMR where low temperatures are necessary.

## Experimental

### Rheological measurements

All rheological measurements were performed using an Anton Paar Physica 301 rheometer, fitted with a chiller to help with the cold temperature measurements. Temperature calibrations were performed between –30 °C and 80 °C before starting the temperature measurements to ensure the correct temperature was being recorded. All data was collected using a vane (ST10-4V-8.8/97.5) and cup geometry (H-24-D) so samples could be prepared in aluminum cups to remove any loading issues. There was a gap distance of 1.5 mm between the bottom of the gel and the cup. A zero force of 0 N was maintained throughout the experiments. Measurements were recorded in triplicate. All measurements were recorded in the linear viscoelastic region of the gels as determined by the strain sweeps, which are recorded first. *G*′ and *G*′′ are determined from the frequency sweeps at 10 rad s^–1^. The yield point is determined at the point at where *G*′ and *G*′′ deviate from linearity in the strain sweep, and the flow point where *G*′′ crosses over *G*′.

#### Strain sweeps

Strain sweeps were recorded from 0.1–1000% strain at 10 rad s^–1^. They were recorded at 25 °C in triplicate. They were then lowered to a temperature few degrees above the freezing point as determined by the freezing point experiments at a rate of 0.5 °C min^–1^ and then a strain sweep was recorded.

#### Frequency sweeps

Frequency sweeps were recorded from 1–100 rad s^–1^ at a strain of 0.5%. They were recorded at 25 °C in triplicate. They were then lowered to whatever temperature at a rate of 0.5 °C min^–1^ and then a strain sweep was recorded.

#### Freezing point determination and temperature stability measurements

*G*′ and *G*′′ and were recorded over time at a frequency of 10 rad s^–1^ and a strain of 0.5%. The temperature was then lowered at a rate of 0.5 °C min^–1^ from 25 °C until there was dramatic increase in *G*′, this indicated that gel had frozen. The rheometer is only calibrated to –40 °C and so that was the lowest temperature the gels were taken down to, so in the case of 60 : 40 glycerol : water the gels did not freeze and so the freezing point could not be determined. To ensure the correct sample temperature a Eurotherm type K thermocouple was also used. This allowed us to check the temperatures past what the rheometer was calibrated to, so that the freezing point of 60 : 40 gels could be determined. In order to reduce the temperature of the rheometer to –40 °C, a water circulator was used at –10 °C, and cardice was used to cool the top of the cup holder.

### Ice nucleation assay

The gels were prepared as stated previously giving ≥15 0.7 μL droplets on each slide. The slide was placed inside a Linkham Scientific cryostage. The cryostage was rapidly cooled to 0 °C at a rate of 50 °C min^–1^ and then held at this temperature for 3 min to allow the temperature of the glass slide and droplets to equilibrate. The samples were then cooled from 0 °C to –49 °C at a rate of 2 °C min^–1^. Ice nucleation was observed using a Veho Discovery VMS-004 Deluxe USB microscope and Veho Microcapture software V 1.3. The experiment was repeated until at least 30 droplet freezing temperatures were recorded. The nucleation of the gels was compared to that of Milli-Q water, the nucleation of which was recorded in the same manner.

## Conflicts of interest

There are no conflicts to declare.

## Supplementary Material

Supplementary information
