# Supplementary material for: Sub-zero temperature mechanically stable low molecular weight hydrogels
Source: J Mater Chem B. 2018 Aug 2;6(44):7274–9. doi: 10.1039/c8tb01668b (PMC6238726; doi:10.1039/c8tb01668b)
Supplement: Supplementary file 1 [file TB-006-C8TB01668B-s001.pdf]

Electronic Supplementary Material (ESI) for Journal of Materials Chemistry B.
This journal is © The Royal Society of Chemistry 2018

Sub-Zero Temperature Mechanically Stable Low Molecular Weight Hydrogels

Alice Fayter,[a] Matthew I. Gibson[a,b] and Emily R. Draper[c*]

a- Department of Chemistry, University of Warwick, Coventry, CV4 7AL, UK.
b- Warwick Medical School, University of Warwick, Coventry, CV4 7AL, UK
c- School of Chemistry, University of Glasgow, Glasgow, G12 8QQ, UK.

*Corresponding author: Emily.Draper@glasgow.ac.uk

SUPPORTING INFORMATION

Synthesis and Materials..S2

Instruments and Procedures..S2

Supplementary Figures..S4

References..S13

Synthesis and Materials

2NapFF was synthesised as previously described.[1] All other materials were purchased from commercial sources and used as received unless otherwise stated. Distilled water was used throughout.

2NapVG,[1] PBI-H,[2] ThFF[3] and ArFF[4] were synthesised as described previously.

Instruments and Procedures

Preparation of Solutions

2NapFF solutions were prepared at 2.5, 5 and 10 mg/mL. The 2NapFF was dissolved in water with one molar equivalent of NaOH and made up to the correct volume with distilled water. The samples were stirred with a magnetic stirrer bar overnight until all the gelator had dissolved. This resulted in viscous transparent solution at pH 9. If the pH was not 9, it was adjust using a small dilute amount of 0.1 M HCl or NaOH.

In the case of the of the glycerol:water solutions, these were all prepared at 5 mg/mL of 2NapFF. The gelator was prepared as described above but the water replaced with 20:80, 40:60 and 60:40 glycerol:water by volume (higher volumes of glycerol did not result in a gel).

Solutions of the other gelators were prepared in the same way at 5 mg/mL of gelator and with 20:80 glycerol:water content.

Preparation of Gels

A slow acidification method was used to gel the solution prepared as described above. This was achieved by adding 8 mg/ml of glucono-δ-lactone (GdL) per mL for the 5 mg/mL, 20:80, 40:60 and 60:40 solutions. For the 10 mg/mL solution, 16 mg/mL of GdL was used and for 2.5 mg/mL solution, 4 mg/mL of GdL was used. The GdL was gently mixed in the solutions by hand to ensure dissolution. The other gelators were gelled using the same method.

For rheological measurements these were prepared in aluminium cups (Anton Paar) and covered with parafilm.

For the ice nucleation assays these were prepared in a 1.5 mL Eppendorf tube and then 0.7 µL aliquots were transferred onto a hydrophobic glass slide (Marienfeld) where they could gel in a hydrated atmosphere overnight.

pH Measurements

pH measurements were recorded using a Hanna instruments pH edge with a FC2020 pH probe which is calibrated using Hanna buffers for a range of pH 2 – 12.

Rheological Measurements

All rheological measurements were performed using an Anton Paar Physica 301 rheometer, fitted with a chiller to help with the cold temperature measurements. Temperature calibrations were performed between -30 °C and 80 °C before starting the temperature measurements to ensure the correct temperature was being recorded. All data was collected using a vane (ST10-4V-8.8/97.5) and cup geometry (H-24-D) so samples could be prepared in aluminum cups to remove any loading issues. There was a gap distance of 1.5 mm between the bottom of the gel and the cup. A zero force of 0 N was maintained throughout the experiments. Measurements were recorded in triplicate. All measurements were recorded in the linear viscoelastic region of the gels as determined by the strain sweeps, which are recorded first. G′ and G″ are determined from the frequency sweeps at 10 rad/s. The yield point is determined at the point at where G′ and G″ deviate from linearity in the strain sweep, and the flow point where G″ crosses over G′.

Strain Sweeps: Strain sweeps were recorded from 0.1-1000% strain at 10 rad/s. They were recorded at 25 °C in triplicate. They were then lowered to a temperature few degrees above the freezing point as determined by the freezing point experiments at a rate of 0.5 °C /min and then a strain sweep was recorded.

Frequency Sweeps: Frequency sweeps were recorded from 1-100 rad/s at a strain of 0.5%. They were recorded at 25 °C in triplicate. They were then lowered to whatever temperature at a rate of 0.5 °C /min and then a strain sweep was recorded.

Freezing point determination and temperature stability measurements: G′ and G″ and were recorded over time at a frequency of 10 rad/s and a strain of 0.5%. The temperature was then lowered at a rate of 0.5 °C/min from 25 °C until there was dramatic increase in G′, this indicated that gel had frozen. The rheometer is only calibrated to -40 °C and so that was the lowest temperature the gels were taken down to, so in the case of 60:40 glycerol:water the gels did not freeze and so the freezing point could not be determined. To ensure the correct sample temperature a Eurotherm type K thermocouple was also used. This allowed us to check the temperatures past what the rheometer was calibrated to, so that the freezing point of 60:40 gels could be determined. In order to reduce the temperature of the rheometer to -40 °C, a water circulator was used at -10 °C, and cardice was used to cool the top of the cup holder.

Ice nucleation assay

The gels were prepared as stated previously giving ≥15 droplets on each slide. The slide was placed inside a Linkham Scientific cryostage. The cryostage was rapidly cooled to 0 °C at a rate of 50 °C/min and then held at this temperature for 3 min to allow the temperature of the glass slide and droplets to equilibrate. The samples were then cooled from 0 °C to −49 °C at a rate of 2 °C/min. Ice nucleation was observed using a Veho Discovery VMS-004 Deluxe USB microscope and Veho Microcapture software V 1.3. The experiment was repeated until at least 30 droplet freezing temperatures were recorded. The nucleation of the gels was

compared to that of Milli-Q water, the nucleation of which was recorded in the same manner.

Supplementary Figures

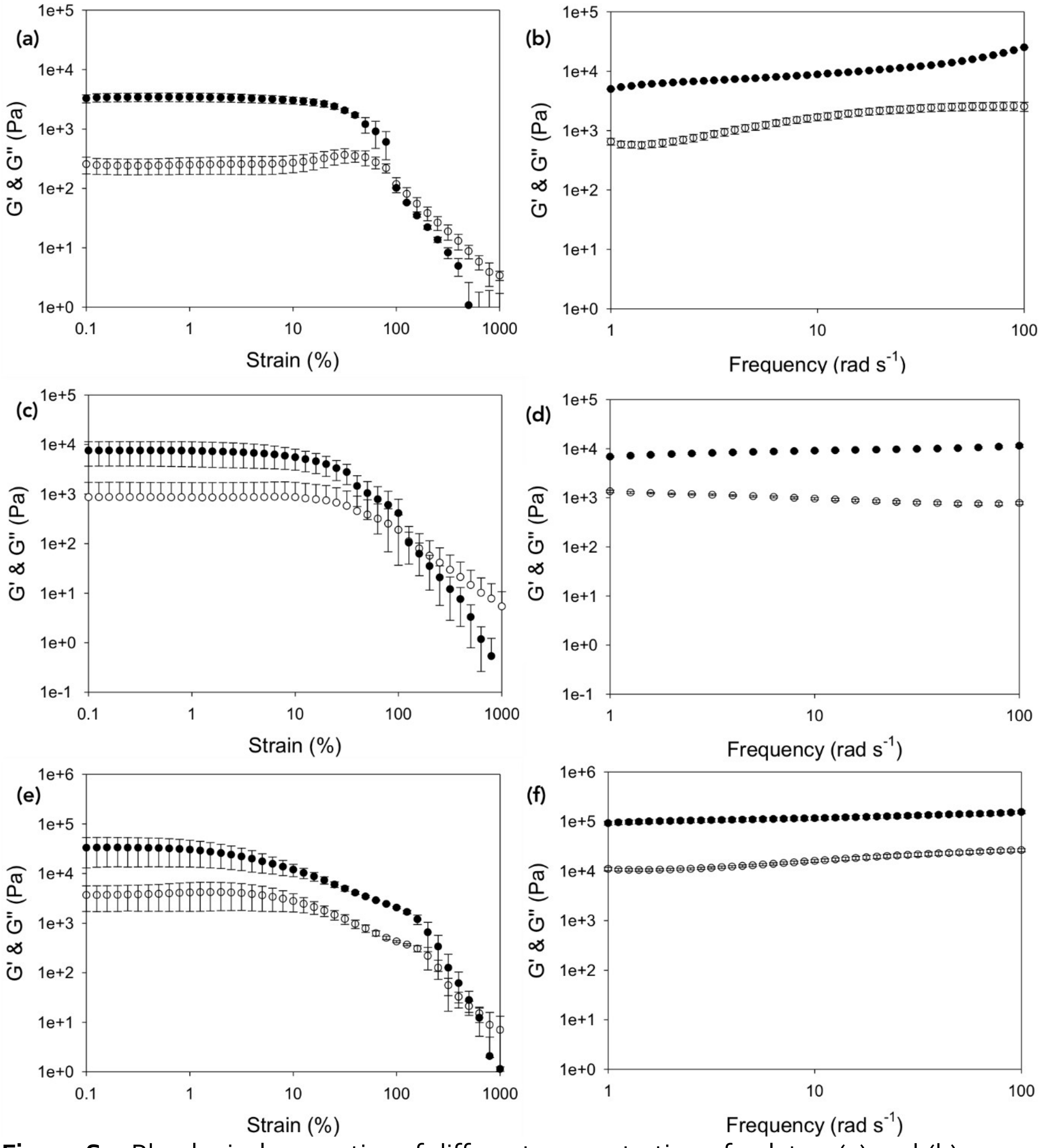

Figure S1. Rheological properties of different concentration of gelator. (a) and (b) are 2.5 mg/mL gels, (c) and (d) are 5 mg/mL gels and (e) and (f) are 10 mg/mL gels. (a), (c) and (e) are strain sweeps performed from 0.1 – 1000% strain at 10 rad/s. (b), (d) and (f) are frequency sweeps performed from 1 – 100 rad/s at 0.5% strain. All measurements were carried out in triplicate at 25 °C and error bars calculated using standard deviation. Solid shapes represent G′ and open shapes represent G″.

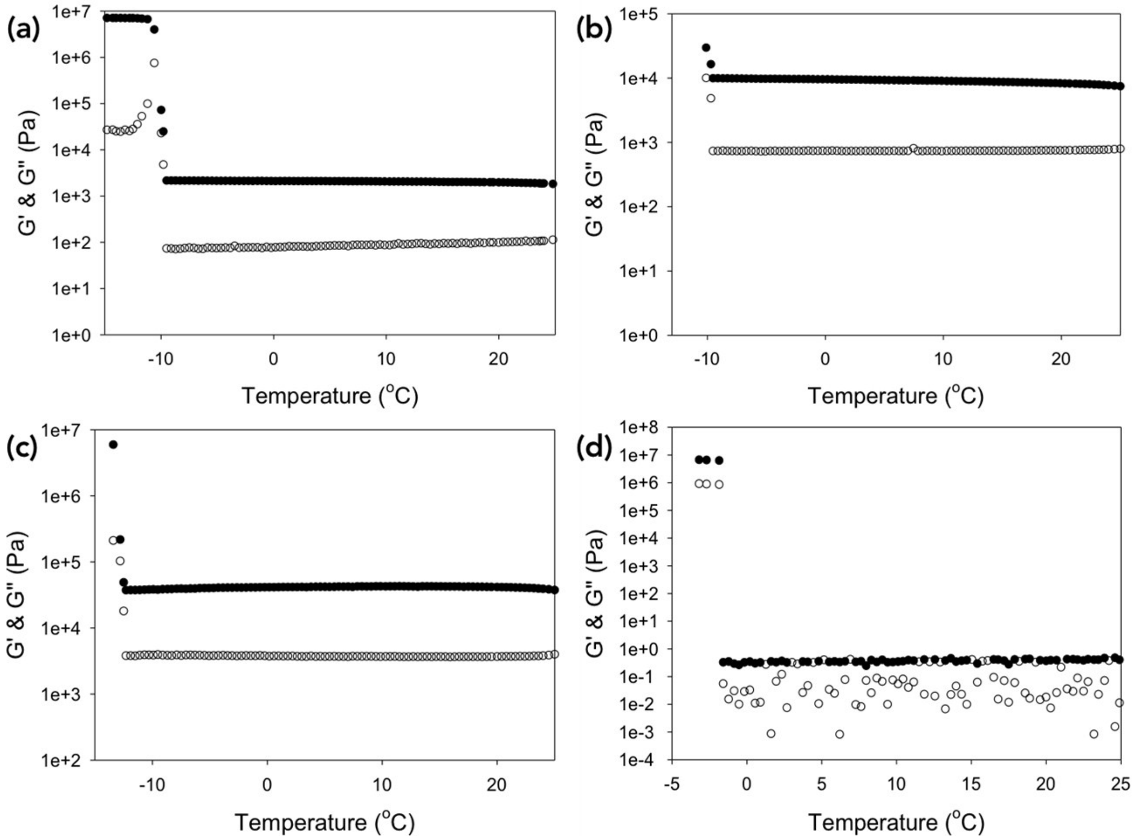

Figure S2. Temperature stability measurements for gels at (a) 2.5 mg/mL, (b) 5 mg/mL and (c) 10 mg/mL. Measurements were recorded from 25 to -15 °C at 10 rad/s and 0.5% strain. Temperatures were lowered at 0.5 °C/min. Solid shapes represent G′ and open shapes represent G″. The freezing point is determined the point at which G′ and G″ dramatically increase.

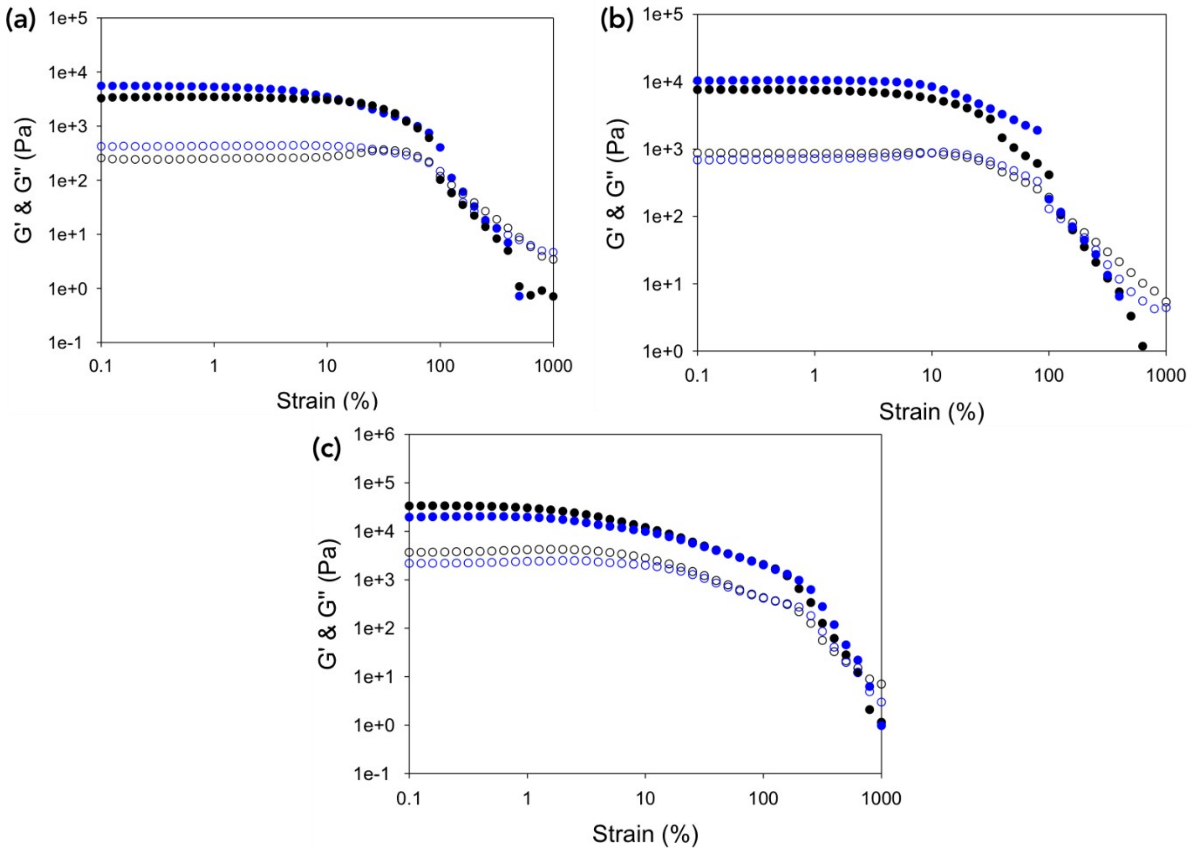

Figure S3. Strain sweeps of gels at (a) 2.5 mg/mL at 25 °C (black data) and at -7 °C (blue data), (b) 5 mg/mL at 25 °C (black data) and at -8 °C (blue data) and (c) 10 mg/mL at 25 °C (black data) and at -10 °C (blue data). No error bars are shown for clarity of the data. Solid shapes represent G′ and open shapes represent G″.

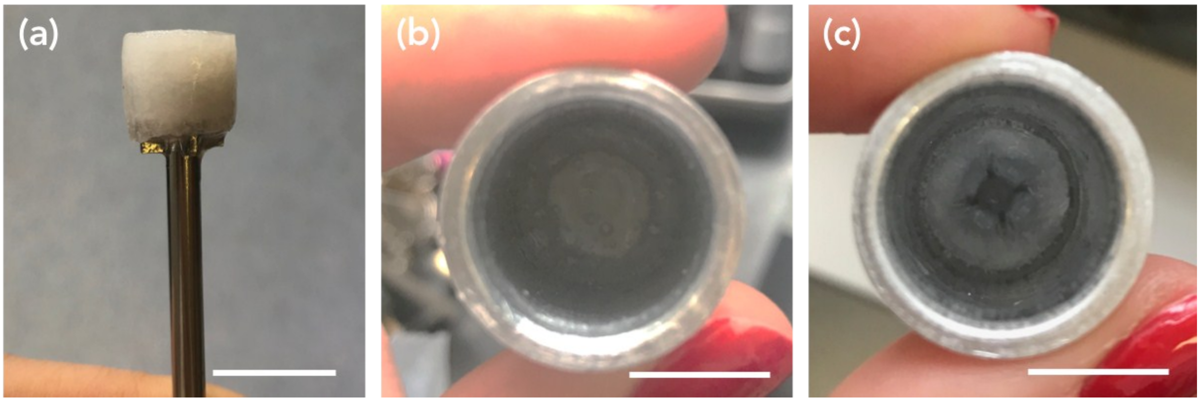

Figure S4. Photographs of (a) a gel at 2.5 mg/mL frozen to the geometry at around -15 °C. (b) a gel at 10 mg/mL at -10 °C and (c) a gel at 5 mg/mL at -10 °C. The scale bars represent 1 cm.

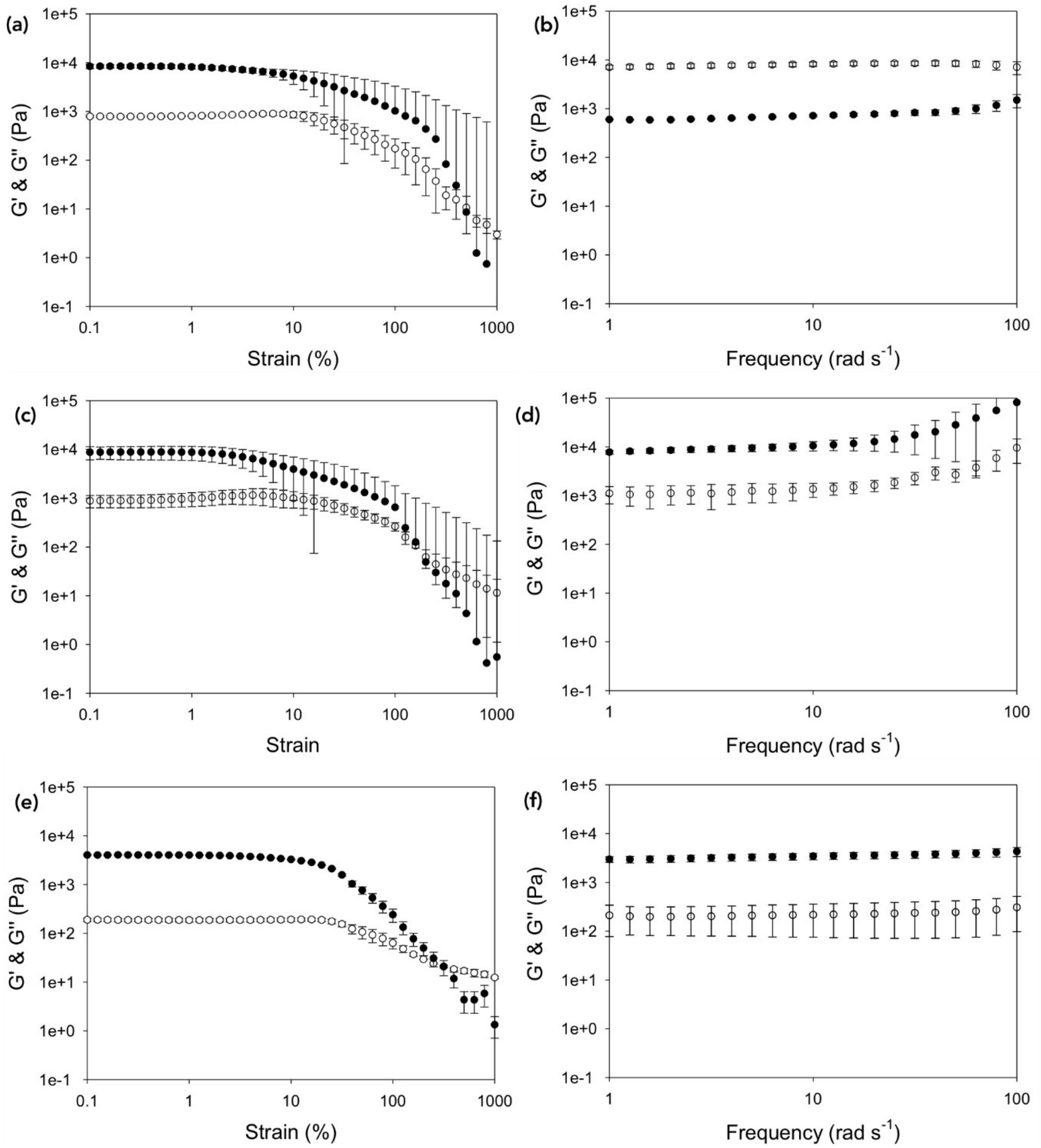

Figure S5. Rheological properties of different glycerol:water ratios. (a) and (b) are 20:80 gels, (c) and (d) are 40:60 gels and (e) and (f) are 60:40 gels. (a), (c) and (e) are strain sweeps performed from 0.1 – 1000 % strain at 10 rad/s. (b), (d) and (f) are frequency sweeps performed from 1 – 100 rad/s at 0.5% strain. All measurements were carried out in triplicate at 25 °C and error bars calculated using standard deviation. Solid shapes represent G′ and open shapes represent G″.

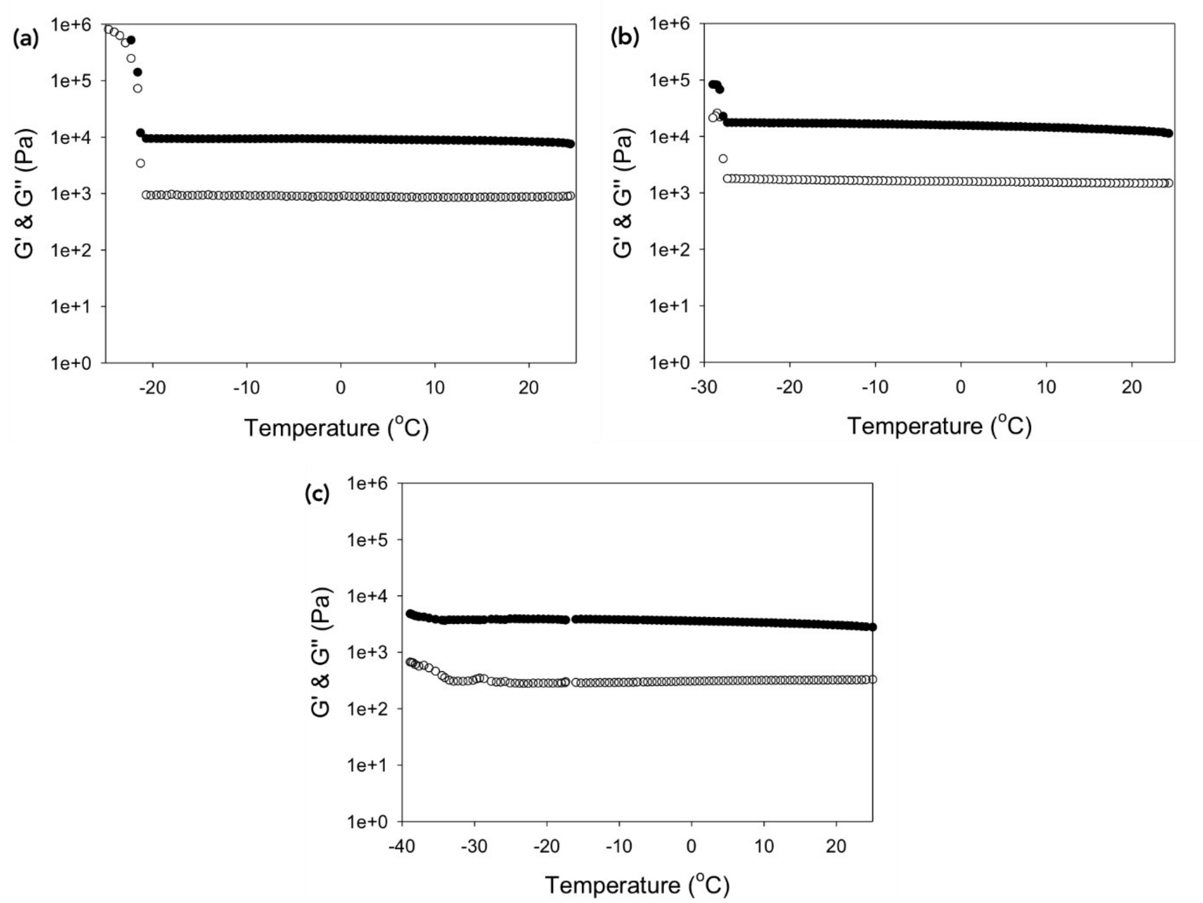

Figure S6. Temperature stability measurements for glycerol:water gels (a) 20:80, (b) 40:60 and (c) 60:40. Measurements were recorded from 25 to -30 °C at 10 rad/s and and 0.5% strain. Temperatures were lowered at 0.5 °C /min. Solid shapes represent G′ and open shapes represent G″. The freezing point is determined the point at which G′ and G″ dramatically increase, for 60:40 gels did not freeze at -40 °C.

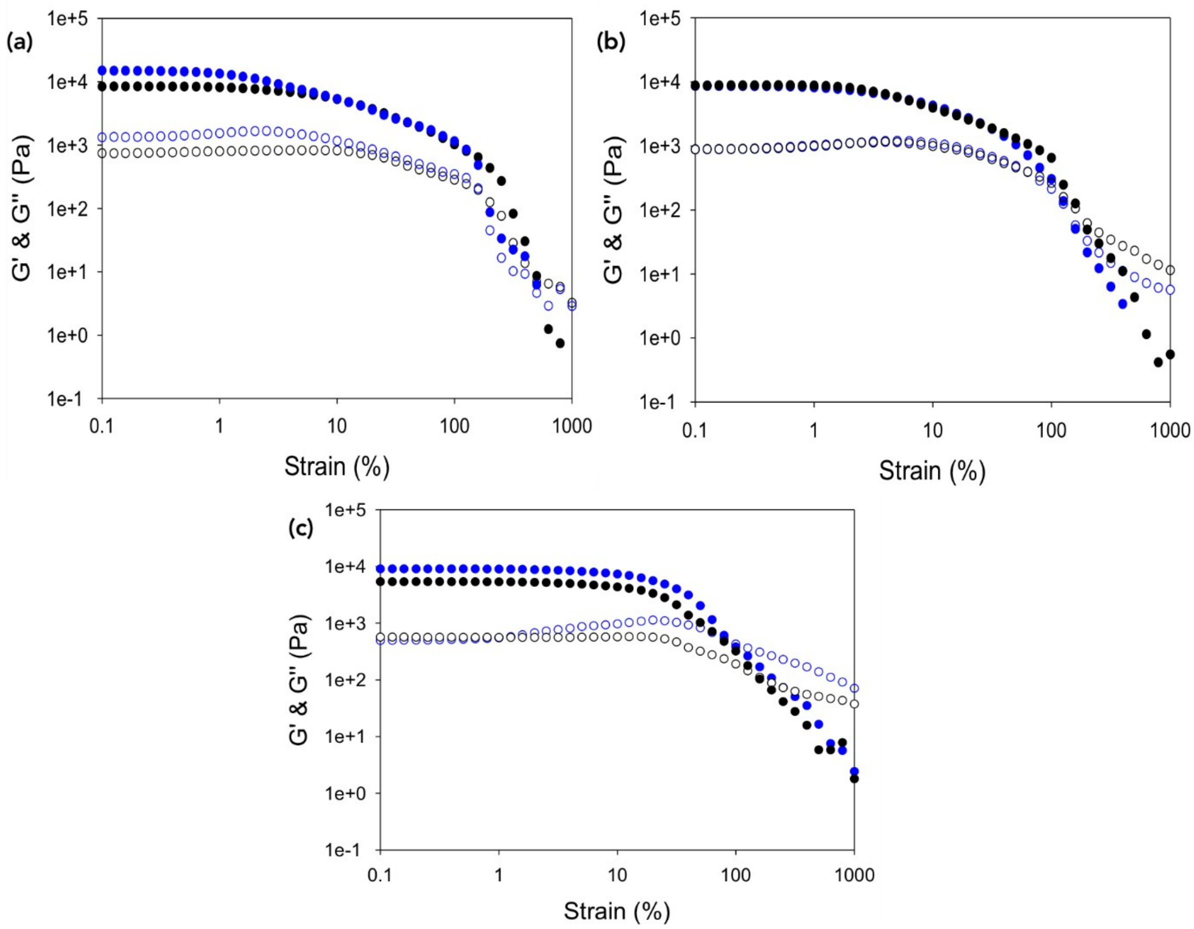

Figure S7. Strain sweeps of glycerol:water gels at (a) 20:80 at 25 °C (black data) and at -20 °C (blue data), (b) 40:60 at 25 °C (black data) and at -25 °C (blue data) and (c) 60:40 at 25 °C (black data) and at -30 °C (blue data). No error bars are shown for clarity of the data. Solid shapes represent G′ and open shapes represent G″.

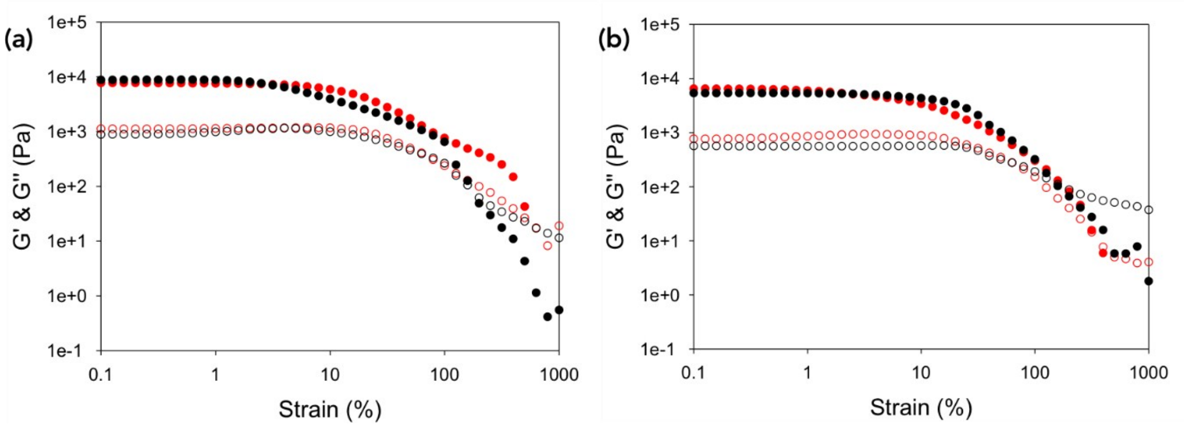

Figure S8. Strain sweeps of (a) 40:60 gels and (b) 60:40 gels, performed at 25 °C. Black data are samples that have not been lowered in temperature. The red data has been lowered left for 2 hours for (a) at -20 °C and so (b) -30 °C, and then the gel has been left to warm up to 25 °C, at approximately 1 °C/min. No error bars are shown for clarity of the data. Solid shapes represent G′ and open shapes represent G″.

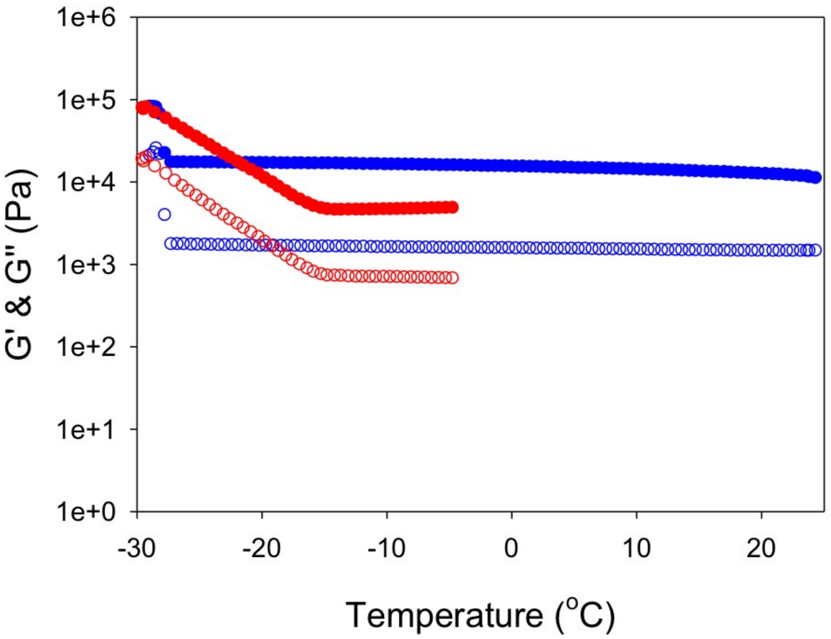

Figure S9. Effect of freezing then warming back up on a 40:60 glycerol:water gel. The temperature was lowered to -30 °C at 0.5 °C/min, which is beyond the freezing point of these gels. The temperature was then increased at the same rate. Measurements were recorded at at 10 rad/s and 0.5% strain. Solid shapes represent G′ and open shapes represent G″, decreasing temperature is the blue data and the increasing temperature is the red data.

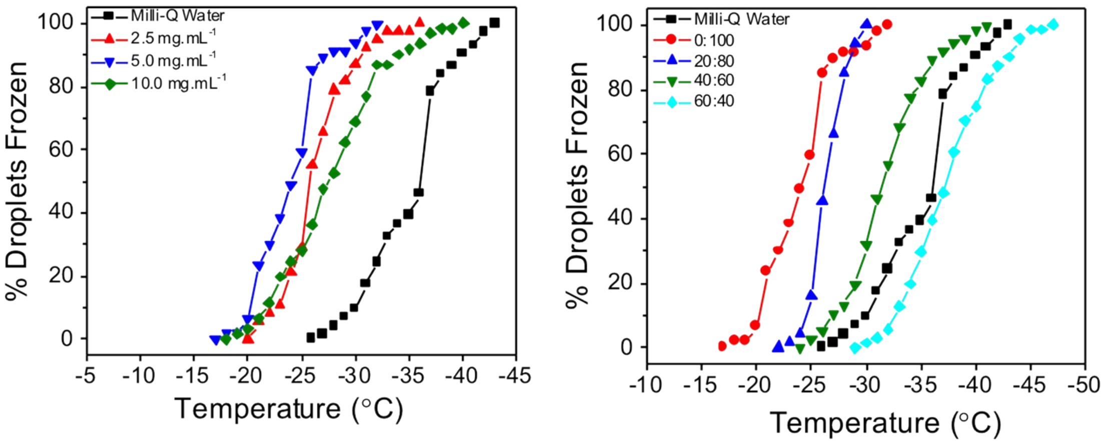

Figure S10. Differential nucleation plots of the droplets frozen as a function of temperature for the different gels studied compared to that of Milli-Q water. The solid lines are provided as a guide to the eyes. (a) Concentration in legend refers to 2NapFF concentration. (b) different ratio Glycerol:Water solutions studied compared to that of Milli-Q water. The solid lines are provided as a guide to the eyes.

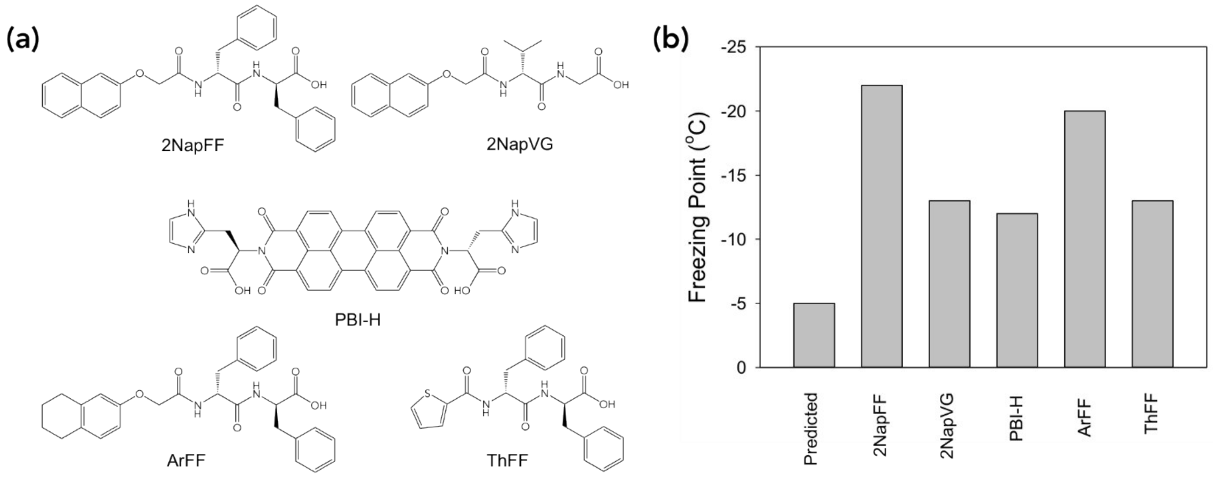

Figure S11. (a) molecular structures of other gelators used in this study. (b) bar chart comparing the freezing points of the different gels prepared at 5 mg/mL of gelator with 20:80 glycerol:water and 8 mg/mL GdL.

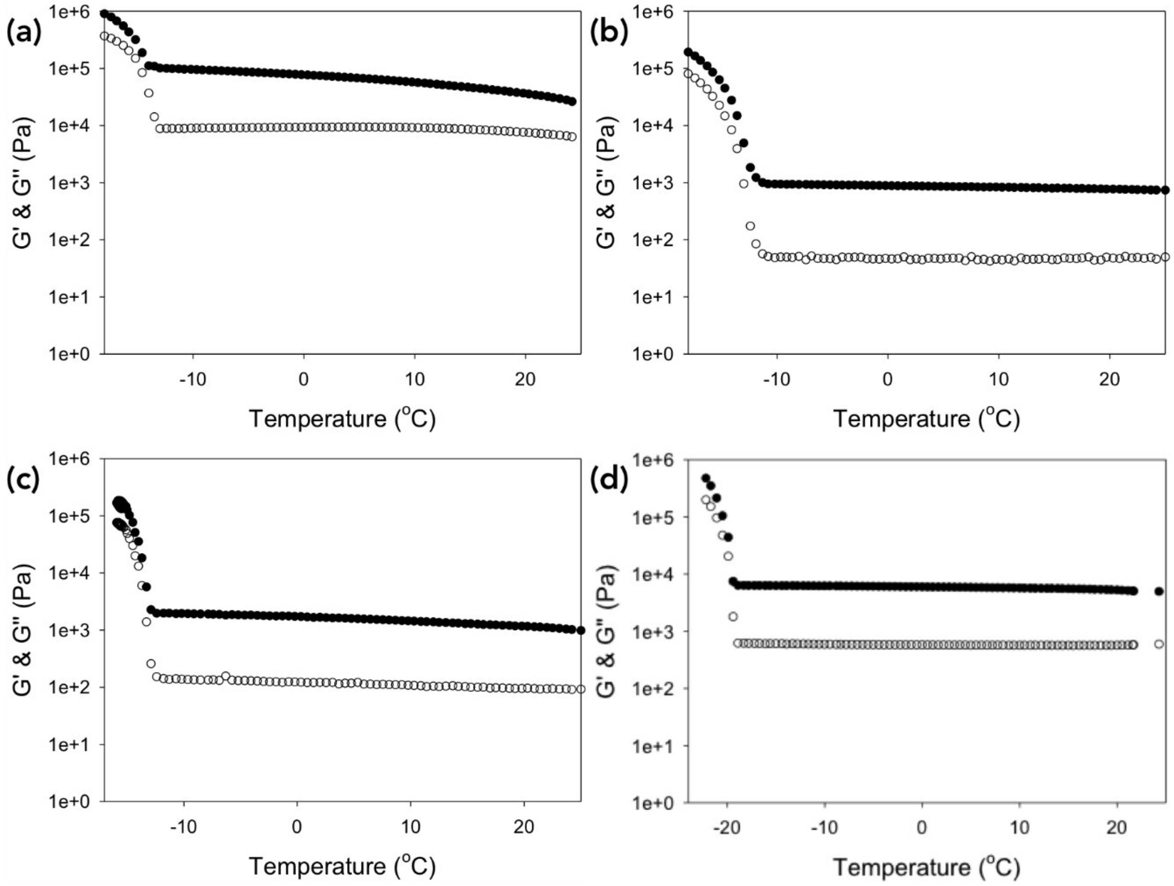

Figure S12. Freezing point measurements for 20:80 glycerol:water 5 mg/mL gels of (a) 2NapVG, (b) PBI-H (c) ThFF and (d) ArFF. Measurements were recorded from 25 till frozen °C at 10 rad/s and 0.5% strain. Temperatures were lowered at 0.5 °C/min. Solid shapes represent G′ and open shapes represent G″. The freezing point is determined the point at which G′ and G″ dramatically increase.

1. L. Chen, S. Revel, K. Morris, L. C. Serpell and D. J. Adams, *Langmuir*, 2010, **26**, 13466-13471.
2. E. R. Draper, J. J. Walsh, T. O. McDonald, M. A. Zwijnenburg, P. J. Cameron, A. J. Cowan and D. J. Adams, *Journal of Materials Chemistry C*, 2014, **2**, 5570-5575.
3. E. R. Draper, T. O. McDonald and D. J. Adams, *Chemical Communications*, 2015, **51**, 6595-6597.
4. J. K. Gupta, D. J. Adams and N. G. Berry, *Chemical Science*, 2016, **7**, 4713-4719.
